# A new Nigerian hunter snail species related to *Enneaserrata* d’Ailly, 1896 (Gastropoda, Pulmonata, Streptaxidae) with notes on the West African species attributed to *Parennea* Pilsbry, 1919

**DOI:** 10.3897/zookeys.840.33878

**Published:** 2019-04-17

**Authors:** A.J. de Winter, Werner de Gier

**Affiliations:** 1 Naturalis Biodiversity Center, P. O. Box 9517, 2300 RA Leiden, The Netherlands Naturalis Biodiversity Centre Leiden Netherlands

**Keywords:** Afrotropical region, Cameroon, computerised tomography, land snail, Liberia, Nigeria, *
Ptychotrema
*, shell morphology, Stylommatophora, taxonomy

## Abstract

*Enneanigeriensis***sp. n.** is described from southeastern Nigeria on the basis of external and internal shell morphology. Following Pilsbry’s formal criteria of a single palatal fold and corresponding external furrow, the new species may be assigned to *Parennea*. *Enneanigeriensis***sp. n.** exhibits substantial similarity with *E.serrata*, a species from Cameroon, in the cylindrical shell shape, crenulate suture, and internal shell morphology, indicating that the two species are closely related. CT scanning confirmed the presence of only a single palatal fold in *E.nigeriensis***sp. n.** and two in *E.serrata*. In spite of this, the Nigerian species is provisionally assigned to *Ennea* rather than *Parennea*, suggesting that the characters used to define *Ennea* and *Parennea* are insufficient to delimit natural groups of species. The holotype of *E.serrata* is examined for the first time since its description in 1896 and a redescription of the species is provided based on the two shells hitherto known.

Study of the original specimens recorded as Ptychotrema (Parennea) sulciferum by Degner from Liberia reveals these to belong to Enneacf.thompsonae. The Nigerian shell recorded by van Bruggen as Ptychotrema (Parennea) aequatoriale proved to be a specimen of Enneacf.perforata. As a result, no species attributable to *Parennea* now appear to be known in West Africa; in contrast, numerous species are known from central and eastern Africa.

## Introduction

The land snail fauna of the Southwest Province of Cameroon (effectively, the area around Mount Cameroon) is comparatively well known due to the pioneering work by von Martens (1876), and the thorough and beautifully illustrated publication by d’Ailly (1896). Additional taxa were introduced in various later studies (Boettger 1905; Degner 1934a; Odhner 1927, 1934; Van Mol 1970; Adam 1981; de Winter 2017).

Still, our knowledge is far from complete. Various species are only known by the original description and often have inaccurate locality data. One example is the streptaxid *Enneaserrata* d’Ailly, 1896, which was described from “Camerunia”. Adolf d’Ailly (1896) attributed the species to the genus *Ennea* H. & A. Adams, 1855, well before Pilsbry (1919) defined and classified *Ennea* as a subgenus of *Ptychotrema* L. Pfeiffer, 1853. Before 1919, *Ennea* was used as a generic heading for numerous, but not necessarily closely related streptaxid species in and outside Africa. Pilsbry (1919) recognised more subgenera and sections within *Ptychotrema*, among others *Parennea* Pilsbry, 1919. Adam et al. (1994) classified *Enneaserrata* as a member of the subgenus Ennea in Pilsbry’s sense based on the original description and illustrations. The present paper appears to be the first study of the holotype shell since its description. Only one more shell has been reported (Jaeckel 1956), but since the specimen was neither described nor illustrated, its identity was not certain.

This investigation was triggered by the discovery of a Nigerian streptaxid species that we initially attributed to *Parennea*, because only a single palatal fold and corresponding external furrow were seen in the shell aperture. In shape and sculpture it strongly resembles the original illustrations of *E.serrata*. However, according to the description by d’Ailly (1896), *E.serrata* is noticeably larger and possesses two palatal folds and corresponding external depressions, typical of *Ennea* in Pilsbry’s (1919) sense. The present paper provides a redescription of *E.serrata* and compares this species with the Nigerian *Parennea*-like specimens described here.

*Parennea* species have been reported from throughout Equatorial Africa. The Democratic Republic of the Congo (formerly Belgian Congo and Zaire) has by far the largest diversity, but the taxon has also been reported from Angola, Cameroon, Ethiopia, Gabon, Kenya, Malawi, Rwanda, Somalia, Tanzania, and Uganda (Pilsbry 1919; Adam and Van Goethem 1978; van Bruggen 1989; Verdcourt 2006; Wronski and Hausdorf 2007; de Winter 2008; Boxnick et al. 2015). Since Adam et al. (1994) transferred the Liberian species Ptychotrema (Parennea) subtusangulatum Degner, 1934, from *Parennea* to *Ennea*, only two records of *Parennea* species from the area between Liberia and the Calabar River (Nigeria) remain. The identity of these two records is addressed here.

The classification of African Streptaxidae is presently still largely based on Pilsbry’s (1919) system, which was tailored for the species of the Congo. Only the genus “*Gulella*” in Pilsbry’s sense has partly been revised (see Rowson and Herbert 2016). Species attributed to *Ennea* and *Parennea* are rather variable in shell shape, size, and sculpture, including that of the protoconch (Adam and Van Goethem 1978; van Bruggen 1989; Adam et al. 1994; de Winter 2008 and unpublished), to the extent that it appears rather uncertain that these taxa constitute monophyletic entities. Shells of species attributed to *Parennea*, and most taxa attributed to *Ennea*, differ profoundly from those of typical *Ptychotrema*. We therefore prefer not to treat these taxa as subgenera of *Ptychotrema*, which would imply a close relationship between them, which in our opinion is very unlikely. Following major syntheses like Zilch (1960) and Schileyko (2002), we here provisionally treat *Ennea* and *Parennea* as two independent genera. However, we realise that some species now attributed to *Parennea* may be more related to some *Ennea* species and vice versa, as was already noted by Adam and Van Goethem (1978). However, the type species of *Ennea* and *Parennea* are conchologically very dissimilar. We therefore prefer to retain them as distinct genera, but we expect that an ongoing study will lead to the recognition of further taxa. Whilst citing opinions of earlier workers in this paper, *Ennea* and *Parennea* are used in the original combinations.

This paper is dedicated to the memory of Professor Chris Omamoke Oke, who passed away in September 2018. Chris was one of precious few terrestrial malacologists in West Africa, working at Benin University, Nigeria, for more than 25 years. His untimely death is a great loss for the field of African non-marine Malacology. We lost a dear colleague, who contributed greatly to this paper.

## Materials and methods

The following acronyms have been used for museum collections:

**MCZ**Museum of Comparative Zoology Harvard University, Cambridge, Mass, USA;

**RMNH**Naturalis Biodiversity Center, formerly Rijksmuseum van Natuurlijke Historie, Leiden, The Netherlands;

**SMNH**Swedish Museum of Natural History, Dept of Invertebrate Zoology, Stockholm, Sweden;

**ZMB**Museum für Naturkunde, Mollusc Collection, Berlin, Germany (formerly Zoologisches Museum Berlin).

Shells were measured and photographed using a Leica M165c stereo microscope with a Leica DFC420 microscope-mounted camera using Leica LAS 4.4 software and Helicon Focus stacking software. The holotype of *E.serrata* (in alcohol) and the holotype of the new taxon from Nigeria were scanned with a Bruker SkyScan1172 Micro-CT scanner (Naturalis Biodiversity Center, Leiden). A voltage of 40 kV was used with a flux of 250 µA, at a full 360° rotation. Medium camera settings were used, with a runtime of approximately 30 minutes. The dry shell of the new taxon from Nigeria was mounted in a gelatin capsule filled with cotton wool, while the holotype of *E.serrata* was mounted in a plastic tube filled with ethanol. Projection images were trimmed and reconstructed using Bruker’s NRecon software. The reconstructed TIFF-images were fully rendered in Avizo 9.4.0 (FEI 2016), using the 3D-volume rendering function and sliced to produce digital cross-sections. Surface models for the CT-scanned specimens are available in MorphoSource (see links in descriptions below).

## Systematic account

### Ennea serrata

Taxon classificationAnimaliaStylommatophoraStreptaxidae

d’Ailly, 1896

**Figure 1.**
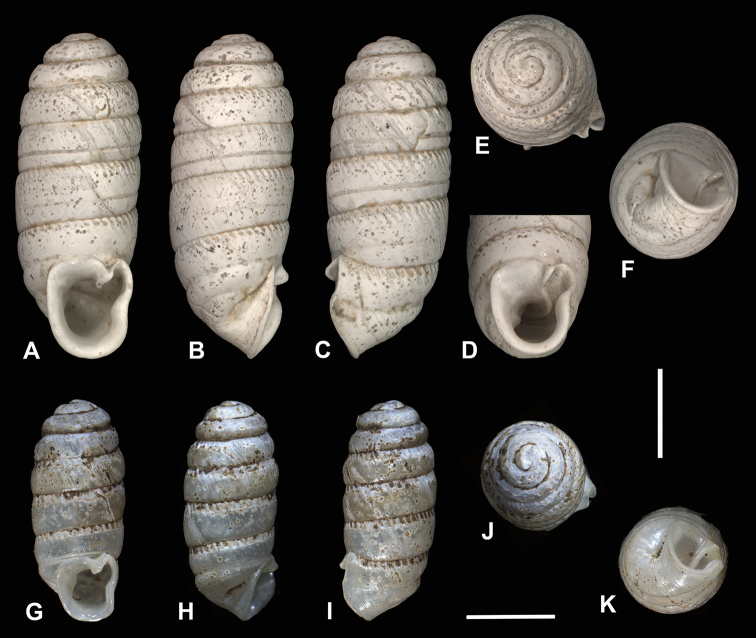
Different views of holotype shells of *Enneaserrata* d’Ailly, 1896 (**A–F**SMNH Type-956) and *E.nigeriensis* sp. n. (**G–K**RMNH.MOL.341194). Scale bar: 2 mm.

**Figure 2.**
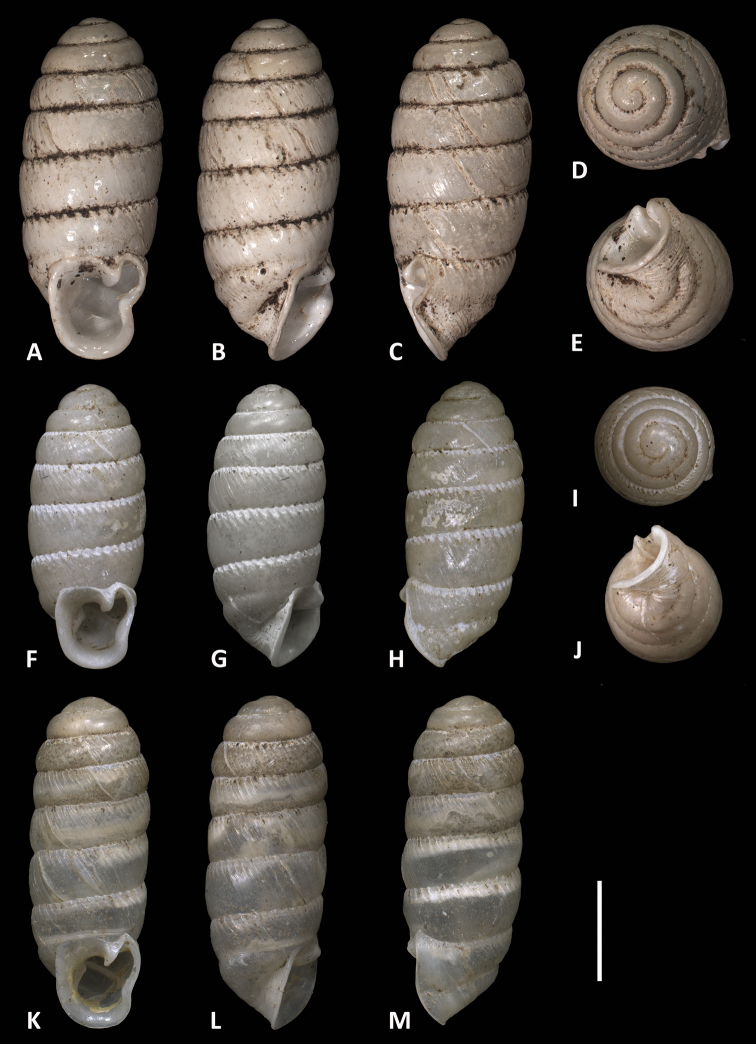
Different views of voucher specimens of *Enneaserrata* d’Ailly, 1896 from Musake Haus, Mount Cameroon (**A–E**ZMB 101777) and *E.nigeriensis* sp. n. (**F–J** and **K–M**RMNH.MOL.341195). Scale bar: 2 mm.

**Figure 3.**
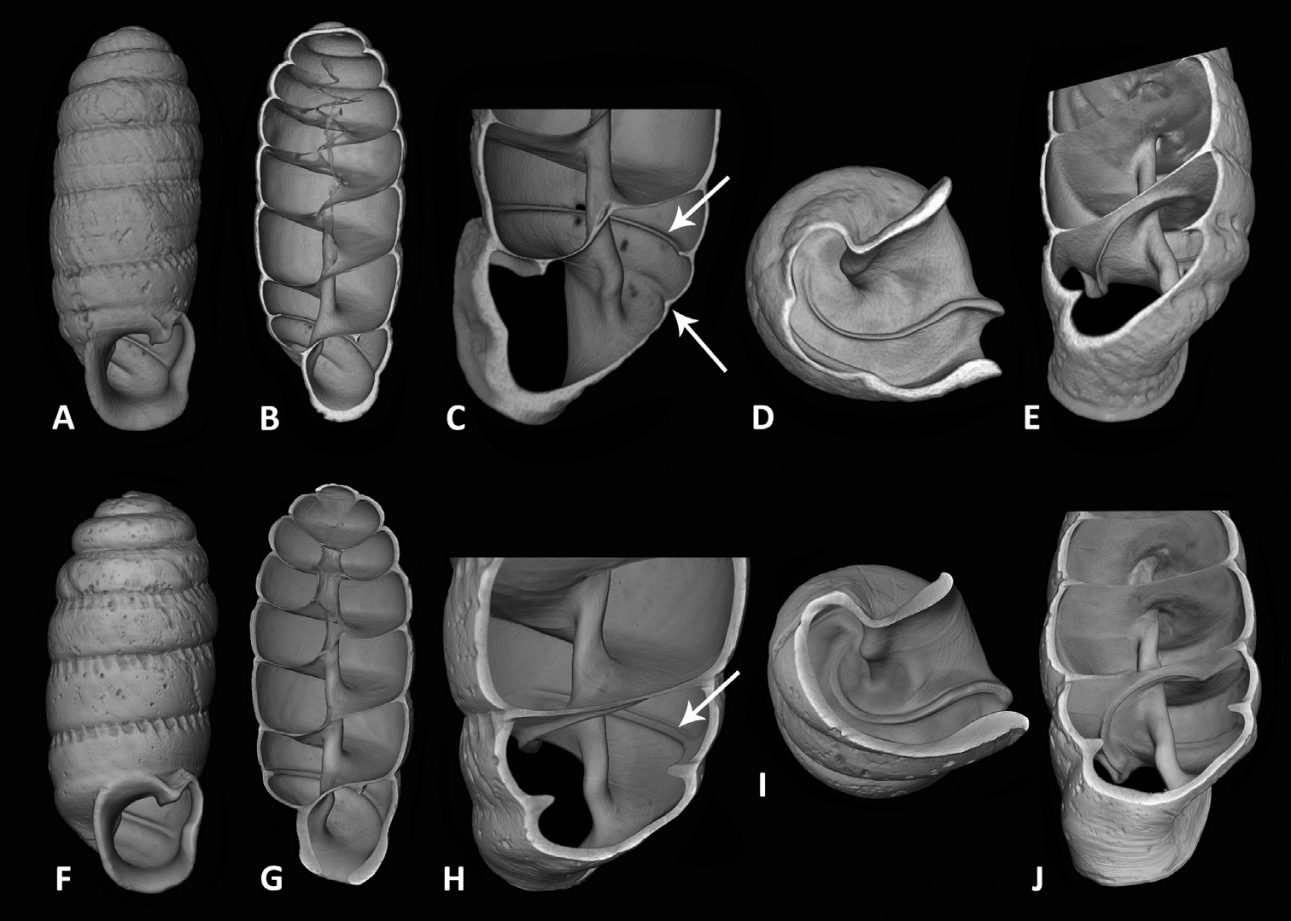
Computerised tomographic images of holotype shells of *Enneaserrata* d’Ailly, 1896 (**A–E**SMNH Type-956) and *E.nigeriensis* sp. n. (**F–J**RMNH.MOL. 341194). **A, F** external shell in frontal view **B, G** internal shell in frontal view **C, H** internal view of body whorl, showing columella, angular lamella, and palatal fold(s) (indicated by arrow) **D, I** view of body whorl ceiling, showing course of angular lamella **E–J** ad-apertural view in body whorl, showing course of angular lamella. Holotype shells are of different dimensions but are scaled to approximately the same size here.

Figs 1A–F , 2A–E , 3A–E

Ennea serrata d’Ailly, 1896: 17, pl. 1, figs 38–41.
Ptychotrema (Ennea) serrata – Jaeckel 1956: 355.
Ptychotrema (Ennea) serratum – Adam et al. 1994: 89.

#### Material examined.

Holotype (SMNH type-956, in ethanol): CAMEROON “Camerunia, ubi?”. Other material: CAMEROON, Kamerunberg, Musake Haus, 1850 m (ZMB 101777/1 dry shell).

The specimen reported by Jaeckel (1956) proved to belong to this species and is the second specimen known. Study of both specimens, together with the internal shell morphology revealed by computerised tomography (CT) scanning, enables some emendation of the original description.

#### Description.

Mean shell height 7.1 mm, with c. 7 whorls, mean shell diameter 2.85 mm (Table 1). Shell cylindrical or subcylindrical, widest at penultimate whorl, apical whorls conical and somewhat more convex than the later ones (see also http://www.morphosource.org/Detail/SpecimenDetail/Show/specimen_id/21851). Protoconch with about 2.5 whorls, protoconch of holotype rather worn, sculpture of the Musake specimen appearing smoothish and glossy, but the eroded suture obscures the transition to the teleoconch; in unworn specimens the transition may be marked by the onset of the subsutural crenulations (as is the case in *E.nigeriensis* sp. n., described below). Umbilicus closed, not perforate as indicated in the original description. Umbilical region below the basal lip with fine, sharp ribs. Teleoconch fairly smooth with irregular growth lines, at some spots the subsutural crenulations extending into irregular, oblique ribs. Suture deep. The original description mentions the presence of very fine decussations at the third whorl (´tertius subtus lineis tenuissimis, oculo nudo haud conspicuis, decussatus’), which were no longer discernable in the holotype, nor in the better-preserved second specimen. Peristome entire, squarish, slightly higher than wide, palatal wall more or less strongly incurved. Columella appears externally as a slightly widened plate, not bidentate as indicated in the original description. CT scanning shows the columella to be a slightly twisted, slender pilaster with a single small dilatation (Fig. 3H, I). Parietal wall emarginate above the angular tooth. Angular lamella starts at the rear side of the strong and protruding angular tooth (an ad-apertural view (Fig. 3J) provides the suggestion of an initial hairpin turn), and coils regularly inwards for about half a whorl, thereby gradually decreasing in height (Fig. 3I, J). Internal wall of body whorl with two palatal folds. The upper fold strong and very long, running for almost an entire whorl from its starting point above the angular tooth towards the palatal lip of the peristome; its termination is just before (and largely obscured by) the swollen palatal lip. The lower palatal fold is less strong and extends for only about half a whorl, ending well before the peristome (Fig. 3F–H). Both folds correspond with external depressions on (parts of) the body whorl.

**Table 1.** Shell measurements of *Enneaserrata* d’Ailly and *E.nigeriensis* sp. n. Abbreviations: SH, shell height; SD, shell diameter; HLW, height of last whorl; PH, peristome height; PD, peristome diameter; W, number of whorls; CT, coiling tightness W:lnH.

		SH	SD	HLW	PH	PW	W	SH/SD	HLW/SH	PH/HLW	PH/SH	PH/PW	CT
* Ennea nigeriensis *	Holotype	5.1	2.2	2.4	1.6	1.4	6.5	2.4	0.5	0.7	0.3	1.1	4.0
	voucher 1	5.3	2.2	2.5	1.6	1.5	6.5	2.4	0.5	0.6	0.3	1.1	3.9
	voucher 2	6.6	2.4	2.9	1.9	1.7	7.6	2.7	0.4	0.6	0.3	1.1	4.0
	voucher 3	5.7	2.4	2.7	1.8	1.6	6.9	2.4	0.5	0.6	0.3	1.1	4.0
	voucher 4	5.3	2.1	2.4	1.6	1.5	6.5	2.5	0.5	0.7	0.3	1.1	3.9
	mean/median	5.6	2.3	2.6	1.7	1.5	6.8	2.5	0.5	0.7	0.3	1.1	4.0
* Ennea serrata *	holotype	7.3	2.8	3.4	2.3	1.9	7.2	2.6	0.5	0.7	0.3	1.2	3.6
	voucher ZMB	6.9	2.9	3.3	2.1	2.0	6.9	2.3	0.5	0.6	0.3	1.1	3.6

#### Ecology.

The Musake specimen was found at 1850 m altitude in the litter layer of a rather moist montane forest rich in ferns, mosses and lichens (Jaeckel 1956).

### Ennea nigeriensis sp. n.

Taxon classificationAnimaliaStylommatophoraStreptaxidae

http://zoobank.org/3607B789-595A-450F-B83B-23EB9C6458EE

Figs 1G–K , 2F–M , 3F–J

#### Material examined.

Holotype (RMNH.MOL.341194) NIGERIA, Cross River State, Agbokim; 5.9833N, 8.7500E, alt. < 100 m; O.C. Oke leg. Other material: 4 voucher shells (RMNH.MOL.341195), same data as holotype.

#### Diagnosis.

Shell small, elongate-cylindrical to elongate ovoid (see also http://www.morphosource.org/Detail/SpecimenDetail/Show/specimen_id/21852). Shell surface with irregular growth lines and coarse subsutural crenulations extending into weak ribs further down the whorl. Shell strongly resembling that of *E.serrata* in shape and sculpture, but differing by the presence of a single palatal fold and corresponding external depression rather than two; in being ca. 20% less wide (and mostly much shorter), with ca. 10% less wide protoconch; and in having tighter coiled whorls.

#### Description.

Shell dimensions: see Table 1. Shell elongate cylindrical or elongate-ovoid, greatest width at penultimate whorl, last whorls less convex than upper whorls, apex subconical. Shell height 5.1 – 6.6 mm with c. 6½ – 7½ whorls. Protoconch large, c. 2¾ whorls, smoothish, protoconch suture simple, transition to teleoconch marked by the onset of the crenulated teleoconch suture. Teleoconch behind the aperture for a short distance with very fine, sharp, distant ribs, rest of the shell with irregular growth lines, the subsutural crenulations at some spots extending into indistinct, low ribs. Suture deep. Umbilicus closed, umbilical region behind the basal lip with fine, sharp ribs. Peristome entire, palatal, basal and columellar wall wide but not strongly reflected, parietal wall with a deep indentation above the strong, protruding angularis. Palatal wall with a blunt mid-palatal swelling. Columellar plate externally appearing as a widened plate with a median protrusion. CT scanning shows the columella as a slightly twisted pilaster with a single small dilatation (Fig. 3C, E). Angular lamella coils regularly inwards for about half a whorl, gradually diminishing in height (Fig. 3C, D). Even more strongly than in *E.serrata*, an ad-apertural view suggests the angular lamella to have an initial hairpin turn which in fact is caused by the lamella being continuous with the angular tooth. A single palatal fold, corresponding with a single, not very deep, external depression, runs for almost an entire whorl from its starting point on the inner wall above the angular tooth towards the palatal peristome lip; its termination is obscured by a swelling of the palatal lip. In transparent shells the internal lamella can be externally seen starting above the peristome as far as the point which the parietal and palatal walls join. This was confirmed by CT scans of the holotype shell, which also demonstrate the absence of a second palatal fold (Fig. 3A–C).

#### Ecology.

The area where the species was collected lies at a rather low elevation, less than 100 m. It may have been collected near the Agbokim waterfall.

#### Remarks.

Although the apertural morphology of the Nigerian shells suggests these to be a member of *Parennea*, they strongly resemble the two known shells of *E.serrata* in overall morphology and are obviously closely related. Pilsbry’s (1919) characters defining *Ennea* and *Parennea* do not appear to delimit natural groups of species. Both taxa exhibit the same variation in shell shape, the shells are either cylindrical or elongate ovoid (cf. Figs 1A, 2A, Figs 1G, 2F). *Enneanigeriensis* is rather variable in shell height, but on average considerably shorter (Table 1). One exceptionally tall specimen is only a trifle shorter than the shells of *E.serrata*, but is substantially (20%) less wide (Fig. 2K–M; Table 1). In the two known shells of *E.serrata*, the upper half of the palatal lip is more strongly receding than in *nigeriensis* shells. The distance between the known localities of the two taxa is c. 200 km. Further collecting, especially of live snails, is required to test the taxonomic decisions taken.

*Enneanigeriensis* superficially resembles *E.okei* (de Winter, 1996), which is a much larger species with a short angular lamella, as well as a quite different internal shell morphology (unpublished CT data).

*Enneanigeriensis* may be attributed to *Parennea*, but differs substantially from other species attributed to this taxon. The species is larger than most *Parennea* species known to date. Only *P.circumcisa* (Morelet, 1885), *P.usambarensis* (Verdcourt, 1958), *P.sperabilis* (Preston, 1910), and *P.connollyi* (Dupuis & Putzeys, 1922) possess shells larger than 5 mm. Judging from the available illustrations and descriptions, *P.usambarensis* from eastern Tanzania is somewhat similar in shape, size and in peristome morphology; its shell has strong ribs that extend over the entire whorl and is more tightly coiled, its palatal tooth seems sharper (resulting in a more sharply protruding lip in lateral view), and the palatal lamella seems to extend less far into the body whorl (Adam and Van Goethem 1978).

##### The identity of Ptychotrema (Parennea) sulciferum sensu Degner in Liberia

**Figure 4.**
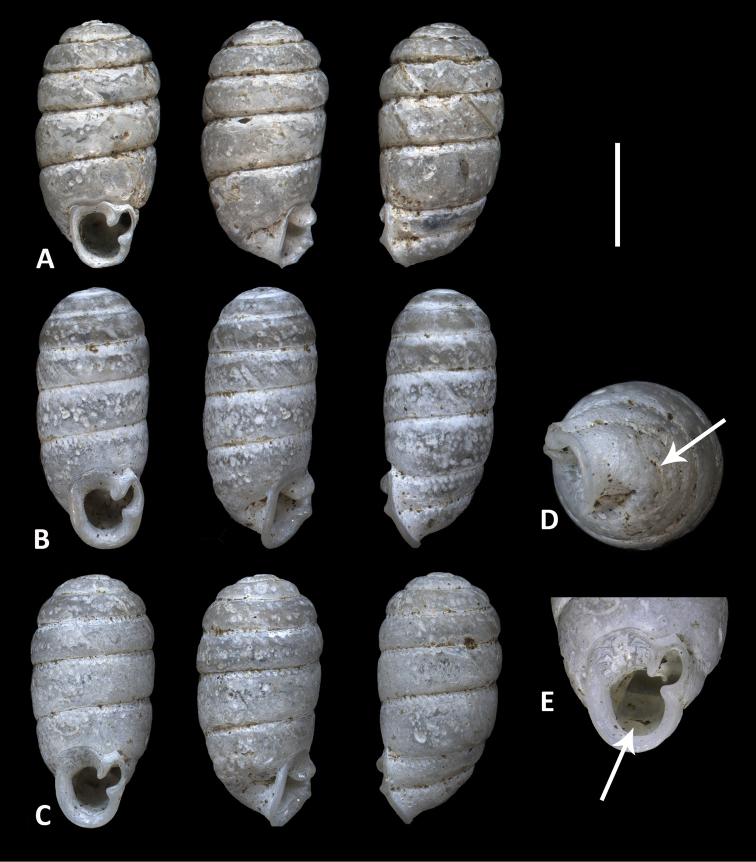
**A–C** Shells of Enneacf.thompsonae Connolly from Du River, Liberia (MCZ 77331), identified and illustrated by Degner (1934b, fig. 8a–c) as Ptychotrema (Parennea) sulciferum. **D, E** enlarged views of body whorl of shell **C** arrows pointing to second external furrow (**D**) and second palatal fold in aperture (**E**). Scale bar: 2 mm (**A–C**).

Degner recorded Ptychotrema (Parennea) sulciferum (Morelet, 1884) from Du River, Liberia and provided line drawings of three shells (Degner 1934b, fig. 8). This species was described from Landana, Cabinda (exclave of Angola), and later recorded from Angola, Uganda (van Bruggen 1989) and Cameroon (de Winter 2008). The Liberian record was not addressed in two major *Parennea* studies (Adam and Van Goethem 1978; van Bruggen 1989). De Winter (2008) refuted Degner’s specific identification and suggested the Liberian shells to belong to an as yet unknown *Parennea* species. In Degner’s drawings the characteristic acuminate apex of *P.sulcifera* is lacking, but they do show a single palatal fold on the last whorl characteristic for *Parennea* species. Degner’s material was collected by Joseph Bequaert and could be borrowed from his collection in MCZ. Study of the three specimens revealed the presence of a second palatal fold and a shallow and easily overlooked additional furrow on the back of the shell (Fig. 4D, E). These characters assign the species to *Ennea* rather than *Parennea*.

An attempt to identify Degner’s specimens with a known *Ennea* species revealed a strong resemblance to shell drawings that Degner provided in another publication (Degner 1934a) as Pt. (E.) elegantulum (L Pfeiffer, 1846). However, in the very same paper in which *P.sulcifera* was reported, Degner (1934b: 372, footnote) corrected his previous identification of Pt. (E.) elegantulum to Pt. (E.) thompsonae Connolly, 1928. Curiously, in the same paper in which Degner (1934b) reported *P.sulcifera* from Du River, he also recorded *E.thompsonae* from that locality. Obviously an error occurred. It seems likely that all shells from Du River belong to the same species. We assume that Degner misidentified the three shells as *Parennea* due to their unusual small size and rather eroded state, largely (but not completely) obscuring the characteristic (for *E.thompsonae*) crenulated suture. We provisionally identify this species as Enneacf.thompsonae, as the shells from Du River are noticeably smaller than the type material of this species from Sierra Leone (see Connolly 1928).

Although Degner (1934b) acknowledged that his previously published description and figure of the genital anatomy of Pt. (E.) elegantulum actually related to *E.thompsonae*, some subsequent authors were apparently unaware of Degner’s correction (but not Adam et al. 1994). For instance, Schileyko (2000: 798, fig. 1040) based his anatomical characterization of *Ennea* on Degner’s (1934a) illustration of *E.elegantula*, thus on *E.thompsonae* (but provided a drawing of a correctly identified shell). Also Binder (1963) illustrated the genital anatomy of *E.elegantula* with Degner’s drawings, but provided shell drawings of what appears to be true *E.elegantula* (as did Daget 2003, who copied Binder’s figures). Remarkably little is known of the type species of *Ennea*, including its anatomy and distribution.

##### The identity of Ptychotrema (Parennea) aequatoriale sensu van Bruggen in Nigeria

**Figure 5.**
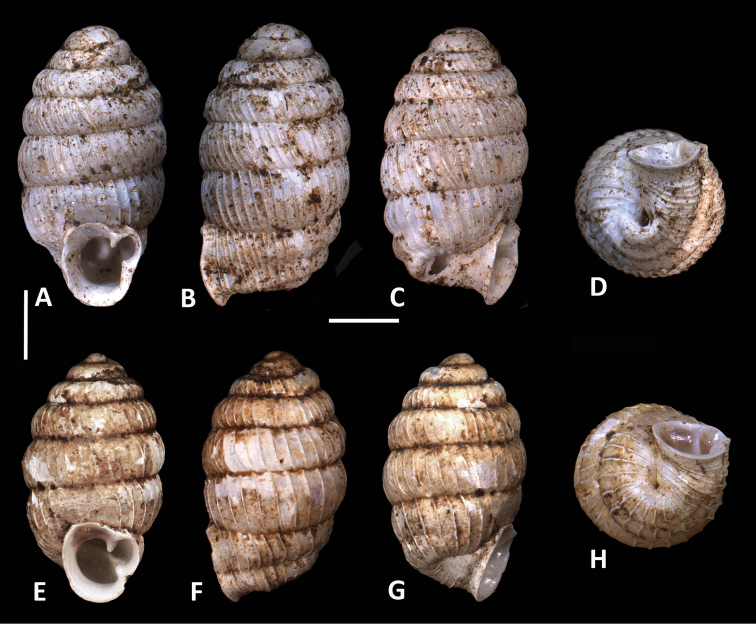
**A–D** Shell of Enneacf.perforata d’Ailly reported by van Bruggen (1991) as Ptychotrema (Parennea) aequatoriale. **E–H** shell of *Parenneaequatorialis* (Pilsbry, 1919) from Yanongo, DR of Congo (RMNH.MOL.273704). Scale bar: 1 mm.

Van Bruggen (1991) identified a single damaged shell from Kwale, Nigeria, as Pt. (Parennea) aequatoriale Pilsbry, 1919, a species originally described from the Ituri forest in the eastern DR Congo. This species was subsequently recorded from various other Nigerian localities (e.g., Oke and Alohan 2006; Oke and Chokor 2009). Comparison of the Nigerian specimen studied by van Bruggen (RMNH.MOL.273794, Fig. 5A–D) with drawings of the holotype shell of *P.aequatorialis* (Pilsbry 1919, fig. 83) and shell drawings in Adam and Van Goethem (1978, figs 20, 21) shows significant differences. We illustrate a specimen *P.aequatorialis* from Yanongo, eastern DR Congo (Fig. 5E–H). The Nigerian shell is similar in size (4.1 × 2.2 mm), but differs in its more elongate shape, much less acuminate apex, wider umbilicus, and less distant ribbing on the teleoconch without spiral sculpture in the interstices. However, the most significant difference is the presence of a second palatal fold and corresponding second external furrow, indicating that this species cannot be a *Parennea*, but by definition, is a species of *Ennea*. The Nigerian specimen resembles the original illustration of *Enneaperforata* d’Ailly, 1896, a not uncommon, but surprisingly little known species that was described on the basis of a single shell from an unknown locality in Cameroon. This species has been reported from various localities in Cameroon (Jaeckel 1956; de Winter and Gittenberger 1998) as well as from Bioko (Germain 1916; Ortiz de Zarate Lopez and Ortiz de Zarate Rocandio 1956; Wronski et al. 2014). We tentatively identify the Nigerian specimen as Enneacf.perforata in view of the variability of the Nigerian and Cameroonian material (unpublished data), and the unclear status of some similar nominal species. A revision of this species group is in preparation.

## Supplementary Material

XML Treatment for Ennea serrata

XML Treatment for Ennea nigeriensis
